# Fluoridated silver nanocomposites for caries management: an in-vitro assessment of the cytological and antibacterial profiles

**DOI:** 10.1186/s12903-025-05691-2

**Published:** 2025-03-09

**Authors:** Marwa M. Essawy, Samar N. Al Achy, Dalia M. Talaat, Magda M. El-Tekeya, Sara Essa, Nouran Nabil, Nour Ammar

**Affiliations:** 1 Department of Oral Pathology, Faculty of Dentistry, Alexandria University, Alexandria, 21521 Egypt; 2 Center of Excellence for Research in Regenerative Medicine and Applications (CERRMA), Faculty of Medicine, Alexandria University, Alexandria, 21521 Egypt; 3 Department of Pathology, Faculty of Medicine, Alexandria University, Alexandria, 21521 Egypt; 4 Department of Pediatric Dentistry and Dental Public Health, Faculty of Dentistry, Alexandria University, Alexandria, 21521 Egypt; 5 Department of Medical Microbiology and Immunology, Faculty of Medicine, Alexandria University, Alexandria, 21521 Egypt

**Keywords:** Cytotoxicity, Gallic acid, Minimum inhibitory concentration, Nano silver, Polyethylene glycol, *Streptococcus mutans*

## Abstract

**Background:**

Silver nanoparticles (AgNPs) have antibacterial properties with potential applications in managing dental caries. Functionalization with fluoride may further enhance AgNPs’ antibacterial efficacy. This study evaluated the impact of fluoridated AgNPs coated with various surface moieties on their safety profile and antibacterial effects against cariogenic bacteria as a potential anti-cariogenic treatment.

**Methods:**

AgNP synthesis followed citrate and gallic acid reduction methods with polyethylene glycol (PEG) and polyvinylpyrrolidone coating. Functionalizing AgNPs with sodium fluoride (NaF) proceeded. Testing the safety of synthesized compounds was done on human gingival fibroblasts and oral epithelial cells. Meanwhile, minimum inhibitory concentration (MIC) determination against *Streptococcus mutans* was executed to verify antibacterial activity.

**Results:**

Gallic-reduced AgNPs revealed higher yielding capacity than citrate-AgNPs. Cytologically, PEGylation reinforced citrate-AgNPs stability and improved IC50 range up to ∼ 4.2 × 10^16^ µg/mL and 64.3 µg/mL on fibroblastic and epithelial lineages. PEGylated AgNPs counteracted the cytotoxicity of free NaF with antagonistic combinational effect of NaF@PEG gallic-AgNPs on gingival fibroblasts. Microbiologically, AgNPs recorded an enhanced antimicrobial activity of ∼ 5.3 ± 2.3 µg/mL averaged MIC against *Streptococcus mutans.* Furthermore, fluoridation of PEG gallic-AgNPs depicted an additive antimicrobial propensity.

**Conclusions:**

This dual action nanoplatform successfully integrates fluoride and silver components, reducing fluoride concentrations to safety range while maximizing silver’s antibacterial properties. Engineered NaF@PEGylated nanosilver formulation represents promising anti-cariogenic strategy that optimizes therapeutic efficacy while maintaining biological safety.

**Supplementary Information:**

The online version contains supplementary material available at 10.1186/s12903-025-05691-2.

## Introduction

Caries is a common chronic bacterial disease with a complex, sequential developmental process, making its prevention a key focus of ongoing antibacterial research [1]. For many years, fluoride has been championed as the most recommended topical agent for caries prevention [2]. Fluoride is widely recognized for its caries preventive and cariostatic properties [1, 2], not only by promoting enamel remineralization but also by exerting antibacterial effects against *Streptococcus mutans* (*S. mutans*). Similarly, silver is often employed in fluoride agents to reinforce its antibacterial potential, while fluoride ions remineralize the tooth structure [3, 4]. Of these agents, silver diamine fluoride (SDF) has shown great success in caries management. However, the black staining of the carious tissue (and possibly soft tissue) caused by the deposition of silver chloride poses a significant disadvantage [5].

The rise of nanotechnology in the medical field has enabled researchers to tackle many challenges. The potential of nanotechnology to enhance the physical and chemical properties of silver allowed the use of nanosilver particles (AgNPs) across numerous biomedical applications [6]. In the dental field, the enhanced antibacterial properties of AgNPs set them as an effective anti-caries agent. The antibacterial and anti-caries effects of AgNPs have been demonstrated in multiple in-vitro studies [5, 6] and clinical trials [7–10], often comparing their results to that of SDF. Silver NPs, in contrast to SDF, do not cause discernable staining of the tooth structure. The reduced staining associated with AgNPs may be attributed to their smaller particle size and different mechanism of action, which involves the direct interaction with the bacterial cell wall and the inhibition of bacterial growth, without forming silver chloride deposits. Investigating the staining potential of several AgNPs’ concentrations using digital spectrophotometry has reported no significant change in color while maintaining a high antibacterial activity [7]. A similar study has also confirmed the lack of staining caused by AgNPs when applied to dentin, with this lack of staining extending for long periods of time, further supporting the aesthetic advantage of this nano compound [8].

Another notable advantage of nanotechnology is the increased surface area-to-volume ratio, which allows for the functionalization of AgNPs with fluoride compounds while ensuring they remain within safe, cytocompatible levels. Thus, by functionalizing AgNPs with sodium fluoride (NaF), studies have demonstrated that fluoride’s antibacterial properties can be significantly enhanced, potentially rivaling and surpassing those of SDF, especially against *S. mutans*, the primary bacterium responsible for dental caries [9–12]. Notably, AgNPs have the advantage of not causing discernable tooth discoloration [8].

However, several factors may influence the antibacterial potential of fluoridated AgNP nanocomposite, including fabrication methods, capping agents, and surface ligands [13]. AgNP synthesis via chemical or green reduction methods may impact nanoparticle stability and integrity. Furthermore, uncoated or electrostatically stabilized (e.g., with citrate) AgNPs may aggregate under high ionic strength conditions [14–16]. Therefore, stabilizing AgNPs covalently with an appropriate capping ligand of long polymeric chains may preclude their aggregation, especially with fluoride conjugation. Among these cappings, polyethylene glycol (PEG) has boosted the stability of AgNPs, helping the preservation of nanoparticles at high ionic concentrations [16]. Moreover, PEG-coated AgNPs (PEG-AgNPs) are less toxic than those coated with other capping agents [17]. Polyvinylpyrrolidone (PVP) is another viable capping ligand that inherits further stability, functionality, and bioactivity to AgNPs [18].

For clinical application, testing the antibacterial capabilities of fluoridated-polymeric-capped AgNPs mandates further verification of their safety profile to human oral tissues, such as gingival fibroblasts and epithelial cells. Despite several clinical trials investigating the anti-caries effects of AgNPs [11, 12, 19, 20], there remains no consensus on the optimal formulation or the most effective coating agent. This study aims to fill this gap by characterizing PEG- and PVP-coated AgNPs synthesized by two methods: green (gallic acid) and chemical (citrate) reduction techniques. The primary goal is to assess how variations in the AgNP physiochemical properties, inherited by different fabrication methods and coating agents, affect the biocompatibility of primary cell lines as measured by their half-maximal inhibitory concentration (IC50). Additionally, the study aims to assess the safety and antibacterial efficacy of the different AgNP formulations against *S. mutans* by determining their minimum inhibitory concentration (MIC). This comprehensive approach will provide insights into the most effective and safe formulation for potential use in dental caries management. Furthermore, it aims to present an optimized formulation that combines fluoride with a silver compound, aiming to reduce the fluoride concentration while enhancing the antibacterial effectiveness of silver.

## Materials and methods

### Synthesis and characterization of AgNP formulae

#### Materials

Sigma Aldrich Chemie GmbH (Germany) supplied silver nitrate (#7761-88-8) and PVP (Mw 40,000, # 9003-39-8), while Alpha Chemika (India) supplied trisodium citrate (#6132-04-3). Gallic acid (#G010110), PEG (Mw 400, #R0125), and NaF (#18-002-01) were donated from Al Andalous Pharmaceutical Industry (Egypt).

#### Methods

The synthesis of AgNPs followed the reduction method, comparing chemical versus green reducing agents and screening two different stabilizers. In the green synthesis technique, 8 mg/mL of gallic acid was used to reduce 10 mg of silver nitrate dissolved in 50 mL DIH_2_O at 1200 rpm stirring (room temperature). Meanwhile, for the citrate-reduced AgNPs, 10 mg of silver nitrate was added to 50 mL heated-DIH_2_O up till the boiling point. Then, 50.5 mg/mL trisodium citrate was added until the color changed to a yellow solution. For polymeric capping of AgNP batches, 0.5% v/w for PVP and 0.5% v/v for PEG were mixed into the 50 mL DIH_2_O before adding silver nitrate [21].

The preliminary detection of synthesized AgNPs was done by UV-Visible spectrophotometer (Nanodrop, DeNovix, DS-11 FX+, USA). The average particle size, polydispersity index (PDI), and particle charge of AgNPs were performed by the dynamic light scattering (DLS) technique using Zeta-seizer (Nano ZS, Malvern Instruments, Worcestershire, UK), with a dilution ratio of 1:6. The morphology and size of AgNPs were determined by transmission electron microscope (TEM; JOEL, JSM-6360LA, JAPAN).

### Determination of the cytotoxicity profile of AgNPs array and NaF

#### Cell lines and materials

Human oral epithelial primary cell culture (OEC, #36063-01) was purchased from Celprogen Inc (CA, USA). Meanwhile, human gingival fibroblasts (HGFs) were isolated and characterized at the Center of Excellence for Research in Regenerative Medicine and its Application (CERRMA) [22]. Each participant signed an informed consent form for the isolation of gingival fibroblasts. Experiments followed the guidelines approved by the Alexandria University Ethics Committee (IRB No. 00010556-IORG0008839).

Both cell lines were cultivated in Dulbecco’s Modified Eagle’s Medium (DMEM high glucose #41965039 for OEC, while low glucose #31885023 for HGFs) supplemented with 10% fetal bovine serum (#26140079) and 0.5% antibiotics (penicillin-streptomycin, #15140122), all supplemented by Gibco (USA). Meanwhile, SERVA Electrophoresis GmbH (Germany) supplied the 3-(4,5-dimethythiazol-2-yl)-2,5-diphenyltetrazolium bromide (MTT, #20395.01). The dimethyl sulfoxide (DMSO, #67-68-5) was available from Thermofisher Scientific (USA).

#### Methods

The cytotoxic effect of different AgNPs (gallic, PVP-gallic, PEG-gallic, citrate, PVP-citrate, and PEG-citrate) on the viability of the HGFs and OEC was evaluated through an MTT assay. In a 96-well culture plate, cells were seeded at a density of 7000 cells/well. The cells were treated with serial concentrations ranging from 1 to 100 µg/mL nanosilver particles, taking untreated cells as controls. After 24 h incubation, 100 µL of MTT (0.05 mg/mL DMEM) were added to each well and incubated at 37 °C for 3–4 h, after which 100 µL/well DMSO was added in darkness. Then, an ELISA reader (Infinite F15 TECAN, Switzerland) quantified the optical density of DMSO-dissolved formazan crystals at 570 nm [23].

Following the same MTT protocol, NaF cytotoxicity on oral epithelial and fibroblastic lineages was executed at serial dilutions starting from 22,600 ppm.

### Mitochondria appraisement

#### Materials

MitoTracker Red CMXRos (#M7512, Invitrogen) for mitochondrial staining and Hoechst 33,342 (#62249, Thermo Scientific) for nuclear staining were supplied from ThermoFisher Scientific (USA).

#### Methods

The initial cytotoxicity results prioritized PEGylated-AgNPs for further assessment of the cellular stress status to confirm their safety profile on the mitochondrial level. The primary cells (HGFs and OEC) seeded (8 × 10^4^ cells/well) on cover slip in 6-well plates were treated with PEGylated AgNPs for 24 h. Then, 100 nM MitoTracker Red were added and incubated for 45 min in a CO_2_ incubator. Visualization by a confocal microscope Leica DMi8 (Leica, Wetzlar, Germany) was done after Hoechst nuclear staining. The log-corrected fluorescent intensity was quantified using imageJ software (1.54f, NIH, USA) [24].

### Determination of MIC on *S. mutans*

The antimicrobial susceptibility testing for the individual AgNP formula and NaFs was determined using the MIC broth method following the reference protocol of the Clinical and Laboratory Standards Institute [25]. *Streptococcus mutans* (a proficiency testing strain) were prepared in brain-heart infusion with 2% sucrose for a concentration of 0.5 on the McFarland scale, verified using colorimetric measurement (Vitek, BioMérieux, Mexico City, Mexico). This concentration is equivalent to 1.5 × 10^8^ colony forming unit/mL. The MIC of the AgNPs panel was determined with a range of 0.25–1024 mg/L in double-fold dilutions prepared according to the Clinical and Laboratory Standards Institute broth microdilution method. Meanwhile, the double-fold serial dilutions of NaF started from 22,600 ppm.

In microdilution plates, each well received 100 µL of serial dilution of compounds (AgNPs panel and NaF) with 100 µL *S. mutans* in Mueller-Hinton broth, respectively. The current research used two control tubes, where 100 µL *S. mutans* (0.5 McFarland) in Mueller-Hinton broth served as a positive control, while a drug in Mueller-Hinton broth functioned as a negative control. All plates were then incubated at 37⁰ C for 24 h in the incubator with a microaerophilic environment (10% CO_2_) in the candle jar method. MIC was determined by visually observing the lowest drug concentration that inhibited visible bacterial growth [26].

### Loading of NaF on pegylated AgNPs panel

After determining the MIC of the different AgNP formulations and NaF with their cytotoxicity profile, PEGylated AgNPs were the most favored for the next fluoridation step to prepare NaF@PEG gallic-AgNPs and NaF@PEG citrate-AgNPs. The approved doses for the loading process were the IC50 of NaF with the MIC of PEG-gallic and PEG-citrate AgNPs. Fluoridation procedures were kept in lightproof black tubes overnight under a vigorous rotation to achieve uniform dispersion of the particles [21].

### Determination of the cellular and microbiological combinational influence of fluoridated AgNP composites

For investigating the combined influence of fluoridated silver nanoplatforms on the cellular response of gingival and epithelial cell lines, primary cells seeded onto 96-well plates were treated with NaF@PEG gallic-AgNPs and NaF@PEG citrate-AgNPs. Cell survival was determined using the MTT assay as described before. Optimal dosages of NaF, PEG-gallic AgNPs, and PEG-citrate AgNPs were then determined by CompuSyn analysis, considering IC50 for each single formula. For combinational index (CI) calculation, NaF was mixed with each formula in an equal constant 1:1 ratio (0.25 to 2 times the IC50 of each drug alone). The CI values insinuate synergism of CI < 1, additive of CI = 1, and antagonism of CI > 1 [27].

Microbiologically, the fractional inhibitory concentration index (FICI) indicated interactions of drug combinations. FICI calculation was through (MIC of drug A in combination/MIC of drug A alone) + (MIC of drug B in combination/MIC of drug B alone). The FICI values imply the following: synergism of FICI≤0.5; additive 0.5< FICI≤1; indifference 1< FICI<4; and antagonism of FICI>4 [28].

### Statistical analysis

The cytotoxicity and microbiological assays were performed in three independent experiments, each in triplicate. The data were collected, tabulated, and statistically analyzed using GraphPad Prism 8.0. The cytotoxicity assays of separate formulae were analyzed using a non-linear regression method, while combinational indices of fluoridated nanocomposites were calculated using CompuSyn analysis. Shapiro-Wilk test was applied to verify the normality of the data. Heteroscedastic data of AgNPs IC50 for HGFs was analyzed by Kruskal-Wallis test, while NaF IC50 for both cell lines was tested by Mann Whitney test. Meanwhile, one-way ANOVA followed by the Tukey multiple comparison test was used to analyze homoscedastic OEC IC50 for AgNPs, MitoTracker Red fluorescence, and MIC results. Two-way ANOVA followed by the Tukey multiple comparison test was used to clarify the statistical significance in viability inhibition of the combinational therapy. For all statistical analyses, α threshold was at 0.05.

## Results

### PEGylation endorses the stability and downsizes AgNPs

**Fig. 1:**
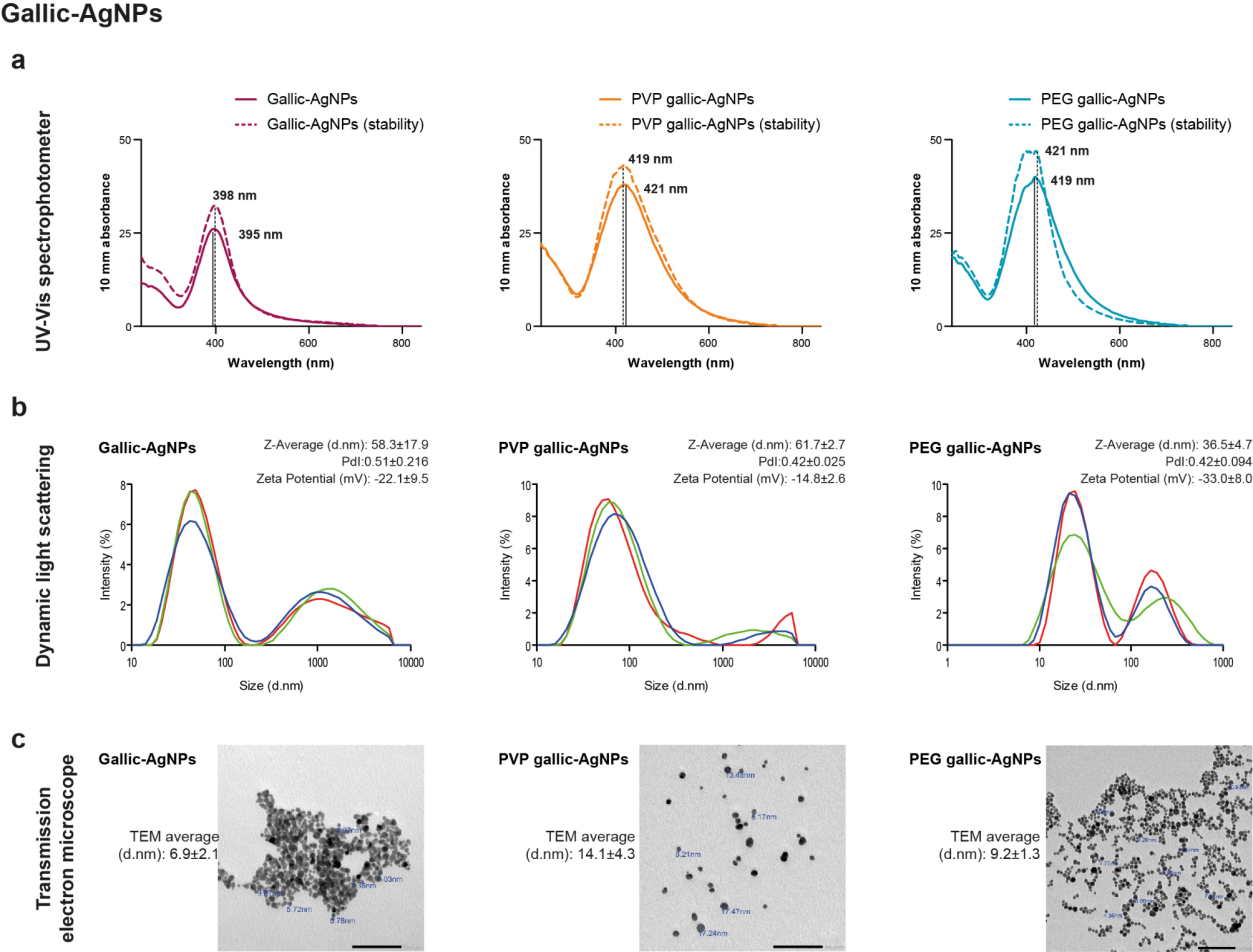
Physio-optical properties of different coated gallic acid-reduced AgNPs. (**a**) UV-Vis spectrophotometer reveals the smooth mono-peaked nanopopulation synthesized by the green reduction method. PEG and PVP capping agents improve the stability of the bared gallic acid-reduced AgNPs, which increases with time. (**b**) DLS displays the non-homogeneity in the nano sizes with multi-peaked curves. (**c**) TEM photomicrographs reveal the pronounced nanoparticle aggregation (clouds) disclosed from the uncapped gallic-AgNPs while moderated by PVP capping

**Fig. 2:**
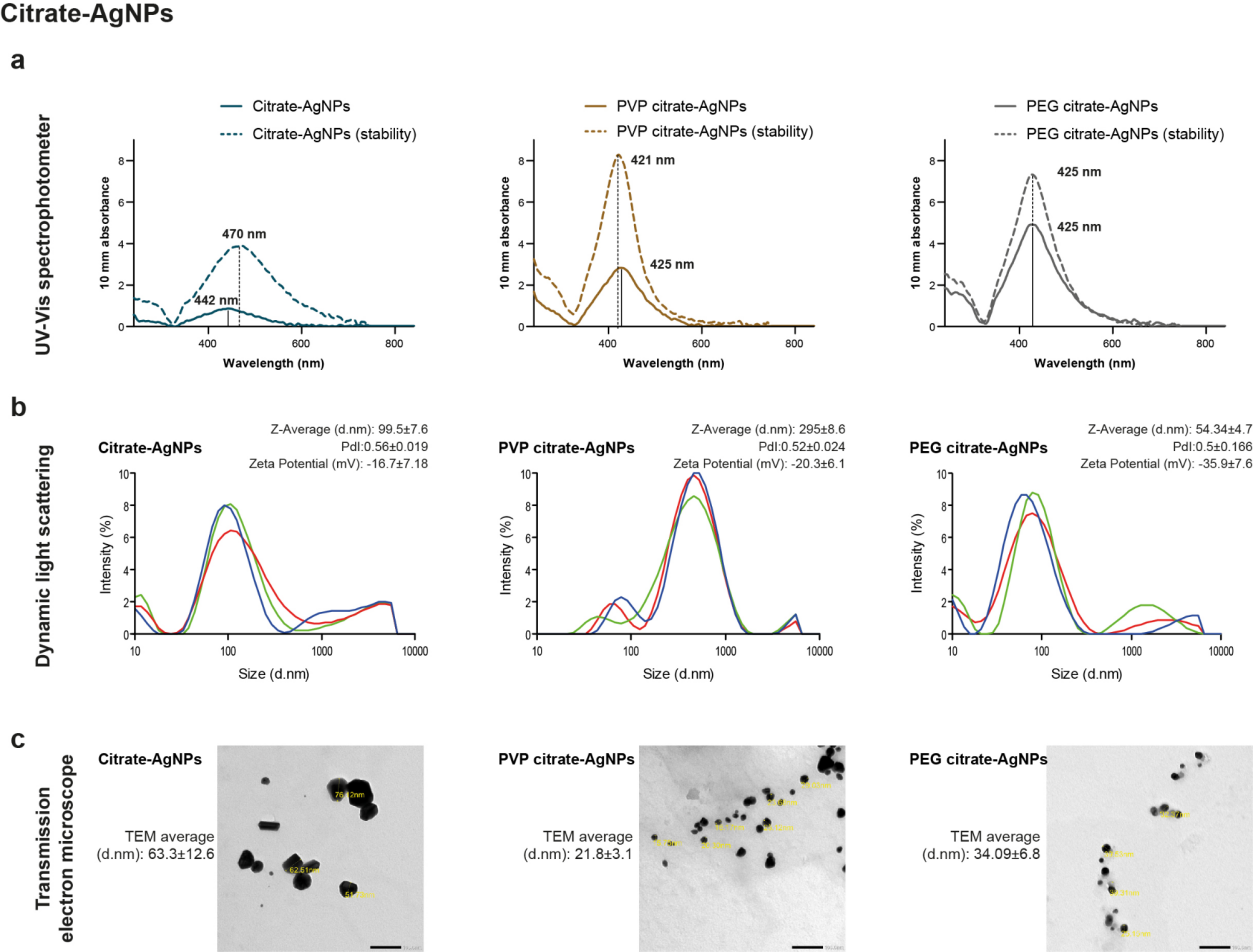
Characterization of the various capped citrate-reduced AgNPs. (**a**) Optically, the uncoated chemically synthesized AgNPs display a broader peak at a lower absorbance value than the capped AgNPs, where PVP and PEG are of added value regarding nano stability and concentration. (**b**) PEGylation of citrate-AgNPs reduces the nanosize by DLS to the smallest size in the same range of the PDI (0.5) as PVP and uncapped nanoparticles. (**c**) TEM visualization indicates the synthesis of monodispersed, widely distributed spherical PVP- and PEG-stabilized AgNPs of more miniature sizes than bared citrate-AgNPs

Synthesis of AgNPs in the present study followed two fabrication methods. Figures 1 and 2 illustrate the optical and physical characterization outcomes of synthesized AgNPs by gallic acid (referred as gallic-AgNPs) and citrate reduction (referred as citrate-AgNPs) techniques, respectively. Both green and chemical methods produced nanopopulations with optical UV-Vis mono-peak in the 419–424 nm absorbance range of AgNPs. However, the gallic acid green reduction method (Fig. 1a) had a higher yielding capacity than the citrate chemical approach (Fig. 2a). Moreover, gallic acid produced AgNPs with smooth, sharp peak absorbed at a narrow UV-Vis range, in contrast to the broader, wide peak of a low absorbance level retrieved from the citrate reduction method.

Upon stabilization of AgNPs, the polymeric coats enhanced the yielding capacity in both synthesis methods. Additionally, their distinguishable effects were evident in improving the stability of AgNPs (Figs. 1a and 2a). By reassessing the optical characteristics of polymeric-capped AgNPs after one month of fabrication, there was a slight shift in the UV-Vis peak, with a coincident stable peak in the PEGylated citrate-AgNPs (Fig. 2a).

Physically, DLS results of both synthesis procedures were of an altered picture rather than the UV-Vis optical single peaks. Nano-Sizer showed multiple peaks, especially in gallic-AgNPs, despite the low PDI recorded around 0.5 (Fig. 1b). Upon coating the green synthesized AgNPs, the aggregation decreased distinguishably with PVP while reduced to a lesser extent upon PEGylation. The added negative charge of PEG to gallic-AgNPs (-33 mV) did not aid the repulsion of nanoparticles from each other due to the smaller 36.5 nm size of PEG-coated batch than the 61.7 nm of PVP-stabilized gallic-AgNPs (Fig. 1b). TEM visualization of the different batches of gallic-AgNPs declared the clumped configuration of the uncoated green synthesized AgNPs in cloud form. Meanwhile, the polymeric stabilized gallic-AgNPs showed an extent of apart distribution, especially the PVP-coated gallic-AgNPs (Fig. 1c).

The results of DLS and TEM for the chemically reduced AgNPs showed that PEGylation of citrate-AgNPs mutually reduced the nano size (to an acceptable range of 54.3 and 34.09 nm, respectively) and increased the zeta potential to -35.9 mV (Fig. 2b). The non-homogeneity in the sizes of PVP-coated citrate-AgNPs was evident in TEM visualization, reflecting Nano-Sizer outcomes that reached 295 nm. Meanwhile, the physical and chemical modifications induced by PEG coating aided the production of widely distributed, monodispersed, homogenous spherical nanopopulation (Fig. 2c).

### The cytocompatibility of pegylated AgNPs versus cytotoxicity of NaF

**Fig. 3:**
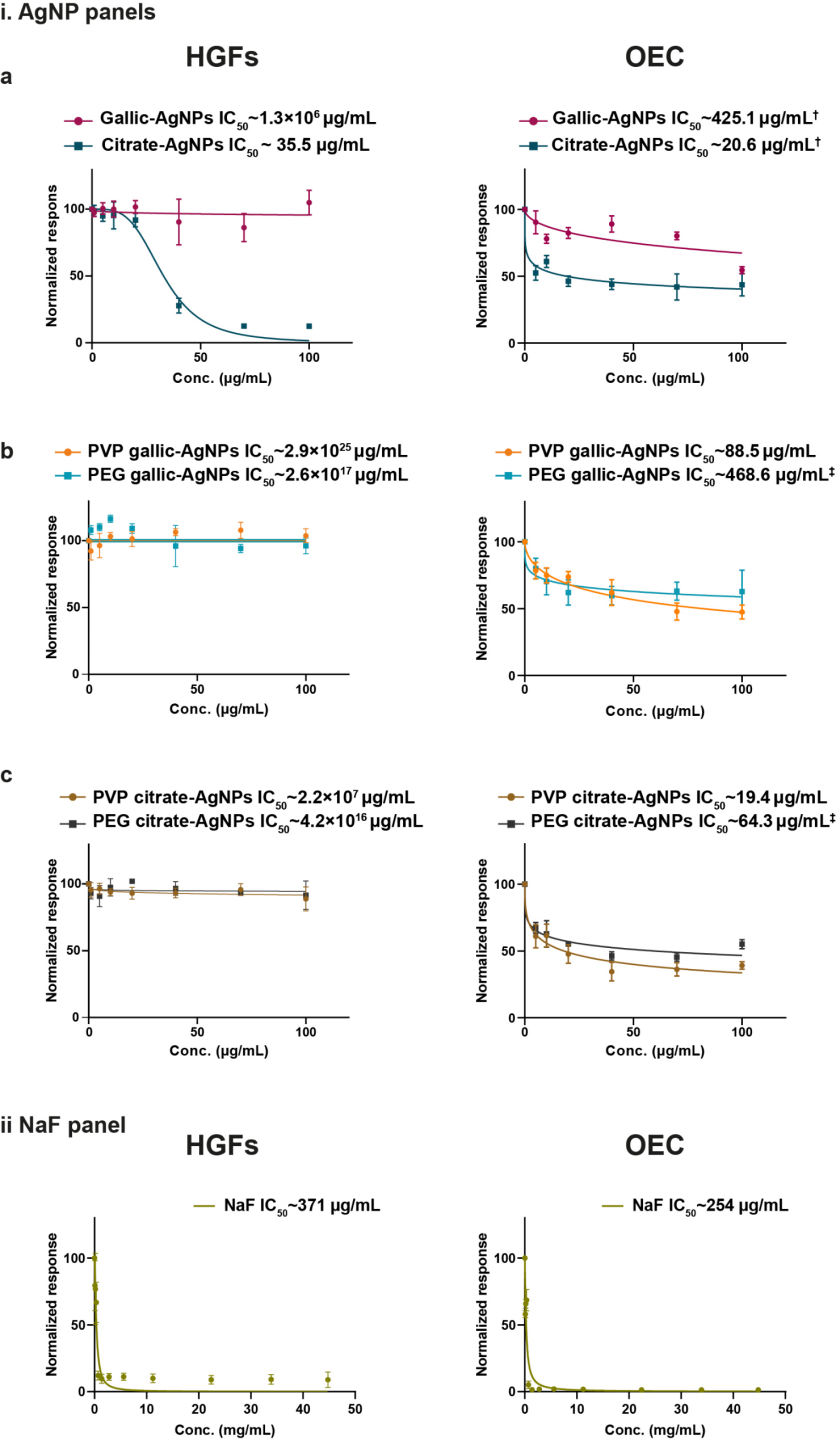
Panel i demonstrates the safety profile of the AgNPs on HGFs and OEC lineages depicted in the non-linear regression curves. (**a**) The uncapped citrate AgNPs reveal higher cytotoxicity towards primary cell lines than the green synthesized AgNPs. (**b** and **c**) PEGylation of the gallic-AgNPs (b) and the citrate-AgNPs (**c**) boost their safety profile, while PVP capping reveals a toxic influence on epithelial cells. Panel ii shows the high sensitization of HGFs and OEC lineages to NaF with an acute drop in cell viability on the dose-dependent curves at very low doses of NaF. The calculated IC50 are the mean of three independent experiments, each of at least triplicates. In AgNP panels, Kruskal-Wallis test reveals insignificance of *p* > 0.05 For HGFs. Meanwhile, one-way ANOVA followed by Tukey multiple comparison test reveals significance of *p* < 0.05 between OEC-treated groups tagged with similar symbols ^†^ and ^‡^. In NaF panel, Mann Whitney test reveals insignificance of *p* > 0.05

As a potential anti-cariogenic candidate, AgNP panels showed a wide range of cytocompatibility when testing their safety profile on HGFs and human OEC. Figure 3 elaborates the dose dependent curves and tags the significant statistical relation in between AgNP batches. Uncapped green synthesized AgNPs were of sound biocompatibility, reaching a plateau schema of dose-related cytocompatibility with high levels of IC50 on HGFs and OEC (∼ 1.3 × 10^7^ µg/mL and ∼ 4.2 × 10^3^ µg/mL, respectively). Meanwhile, chemically reduced citrate-AgNPs displayed adverse toxic effects when applied uncoated, dropping the IC50 to ∼ 35.5 µg/mL and ∼ 20.6 µg/mL for HGFs and OEC, respectively (Fig. 3 panel i.a.).

Stabilizing AgNPs with PEG modulates their cytological response, elevating the IC50 10-fold, especially in fibroblast cell lineage. In respective AgNP panels, PEGylated gallic- and citrate-AgNPs recorded an extraordinary jump of IC50 to ∼ 2.6 × 10^17^ µg/mL and ∼ 4.2 × 10^16^ µg/mL on HGFs. Vulnerable epithelial cells showed a steadiness in the IC50 towards PEGylated green synthesized AgNPs and a slight dose rise for PEG citrate-AgNPs. On the other hand, the PVP-coated nanoparticles showed an inconsistent cytological response, where the IC50 in epithelial cells lowered dramatically with a surge of IC50 in the resistant HGFs, regardless of the fabrication techniques (Fig. 3 panel ib and ic).

Figure 3 (panel ii) displays the cytological responses to NaF. The anti-cariogenic agent declares an extraordinary sensitivity of both cell lineages at a very low dose, reaching an early halting of the metabolic activity of HGFs and OEC on the initial doses of NaF. An acute drop in the dose-dependent curves was at ∼ 371 µg/mL and 254 µg/mL for HGFs and OEC, respectively (with no statistical difference between cell lines, *p* > 0.05). The equivalent 371 ppm and 254 ppm cytotoxic doses were questionable to exert their anti-cariogenic impact as efficiently as the globally used 22,600 ppm, which posed the research question for testing the antimicrobial efficacy of the low doses of NaF together with the novel AgNPs candidate.

**Fig. 4:**
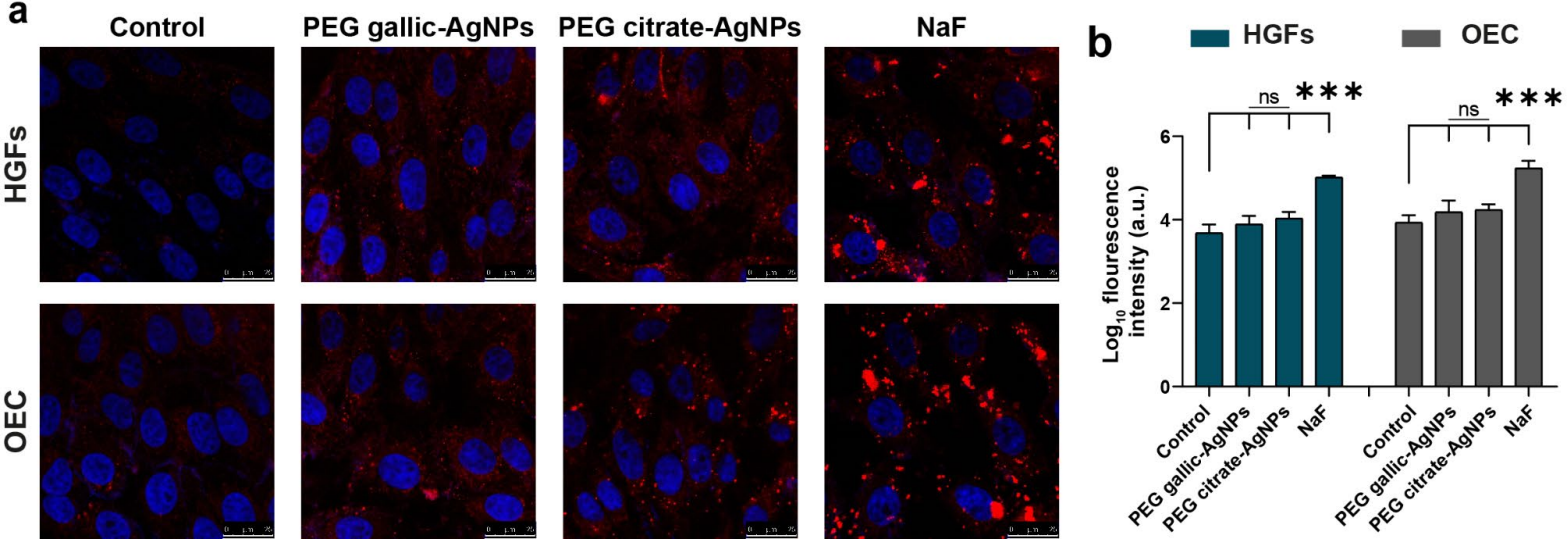
Assessment of mitochondria stress in PEGylated AgNPs treated primary cells versus NaF. (**a**) Representative MitoTracker Red-stained confocal microscopic images (scale bar = 25 μm) show the intense signals in the stressed NaF-treated cells against the mild fluorescence retrieved from cellular emolliated PEGylated AgNP panels. (**b**) The bar graphs for corrected log fluorescence intensities, where*** of *p* < 0.0001 marks the significant results of one-way ANOVA followed by multiple comparison test. Data are the mean of three random microscopic fields captured per each of the three wells for each group

For cellular assessment of the cytocompatibility of the PEGylated AgNP panels (PEG gallic-AgNPs and PEG citrate-AgNPs), the MitoTracker Red stain displayed insignificant fluorescent signals similar to the control intensities in both cell lines (*p* > 0.05), indicating minimal mitochondrial stress. Meanwhile, NaF-treated primary cells revealed intense fluorescence (*p* < 0.0001), implying active mitochondrial sensitization, a key marker of apoptosis (Fig. 4).

### The sturdy antimicrobial efficacy of AgNPs and NaF

Validating the antibacterial potentiality of the tested AgNP panels and NaF, they showed potent antimicrobial properties against the cariogenic *S. mutans* at MIC values lie within the safe range of their cytological IC50 cutoff (Table 1). With a serial dilution of NaF starting from the anti-cariogenic dose of 22.6 mg/mL (22,600 ppm), *S. mutans* was strongly sensitive to the anti-cariogenic candidate at extremely low doses, reaching 170.6 ± 73.9 µg/mL.

**Table 1:** MIC values of tested compounds

	MIC range (µg/mL)	Mean (µg/mL)
NaF	128–256	170.6 ± 73.9
Gallic-AgNPs	4.0–8.0	5.3 ± 2.3 ^a^
PVP gallic-AgNPs	4.0–8.0	5.3 ± 2.3 ^a^
PEG gallic-AgNPs	4.0–8.0	5.3 ± 2.3 ^a^
Citrate-AgNPs	4.0–4.0	4.0 ± 0.0 ^a^
PVP citrate-AgNPs	4.0–8.0	5.3 ± 2.3 ^a^
PEG citrate-AgNPs	8.0–16.0	10.6 ± 4.6 ^a^

^a^ Denotes significant difference of *p* < 0.0001 in the MIC between the tested AgNPs batches and NaF analyzed by one-way ANOVA analysis

Silver NPs revealed close MIC ranges, with a robust sensitivity of *S. mutans* to low doses of AgNPs. The green synthesized AgNPs, whether uncapped or capped gallic-AgNPs, showed a consistent MIC of ∼ 5.2 ± 2.3 µg/mL. Meanwhile, chemically reduced AgNPs exhibited a small range of MIC variation, where citrate-AgNPs recorded antibacterial effects at ∼ 4.0 ± 0.0 µg/mL that increased 2.7-folds with PEGylated citrate-AgNPs, registering MIC of ∼ 10.6 ± 4.6 µg/mL. The antimicrobial efficacy of PVP-capped citrate-AgNPs returned to the MIC levels of the green fabricated AgNPs. The recorded MIC was of significant difference between NaF and AgNP batches (*p* < 0.0001). Meanwhile, MIC in-between AgNPs was insignificant *p* > 0.05. Supplementary Table 1 displays the detailed results of the half-maximal cytological and minimal microbiological inhibitory doses of AgNPs with their relevant used extinction coefficient.

The steadiness in the physio-optical properties combined with the promising cytocompatibility and antimicrobial profile of PEGylated AgNPs (green- and chemically-reduced AgNPs) led the coming step to focus on the combination effect of NaF with PEGylated AgNPs cytologically and microbiologically.

### The synergistic combined effect of pegylated nanocomposite (NaF@PEG-AgNPs)

**Fig. 5:**
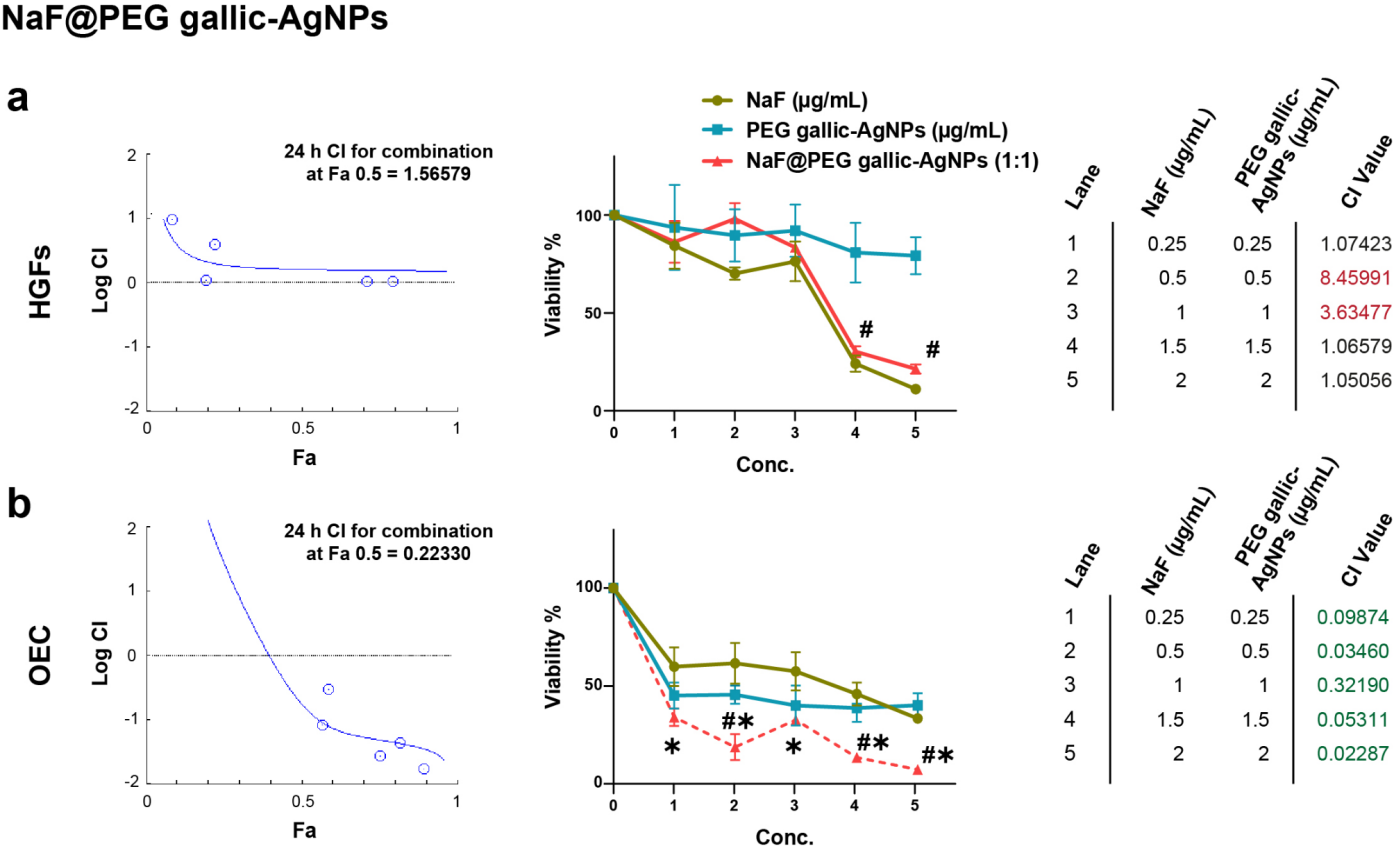
The cytological combinational index (CI) of NaF@PEG gallic-AgNPs on primary cell lines. (**a**) In gingival fibroblasts, the constant 1:1 (NaF: PEG gallic-AgNPs) combination ratio exhibits an antagonistic impact at all doses. Meanwhile, the epithelial cells (**b**) are highly sensitized to the combinational nanoplatform, inducing a significant synergistic impairment of cell viability than the treatment alone. CompuSyn (0.1) results of CI > 1 indicate an antagonistic effect, CI = 1 points out additive impact, while CI < 1 denotes synergistic effects. In the line graph, *points out the significance (*p* < 0.001) of combined nanoplatform with NaF, while # marks the significant relation (*p* < 0.001) with PEG gallic-AgNPs. Viability inhibition (%) data are the mean of three independent experiments, each of triplicates analyzed by tow-way ANOVA followed by Tukey multiple comparison test

**Fig. 6:**
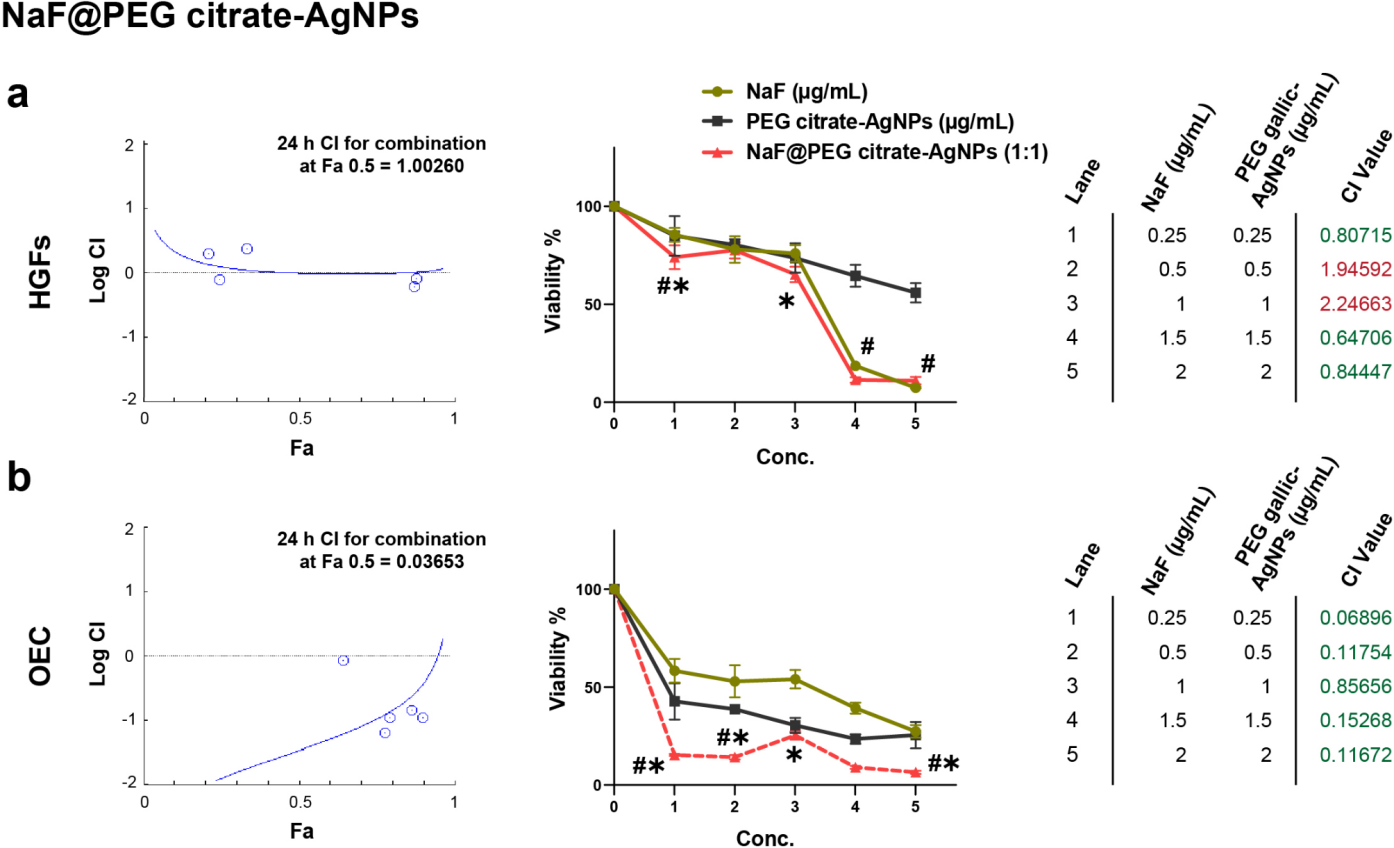
The combinational index (CI) of the NaF@PEG citrate-AgNPs on HGFs and OEC cells. (**a**) In HGFs, the combination of NaF in a constant ratio of 1:1 with PEG citrate-AgNPs induces a significant inhibitory effect on cell viability, fluctuating between additive and synergistic influences. (**b**) In OEC, the nanocomposite reveals a consistently synergistic effect along all doses. CompuSyn (0.1) results of CI > 1 indicate an antagonistic effect, CI = 1 points out additive impact, while CI < 1 denotes synergistic effects. In the line graph, * points out the significance (*p* < 0.001) of combined nanoplatform with NaF, while # marks the significant relation (*p* < 0.001) with PEG citrate-AgNPs. Viability inhibition (%) data are the mean of three independent experiments, each of triplicates analyzed by tow-way ANOVA followed by Tukey multiple comparison test

A forward step in optimizing the anti-cariogenic nanoplatform, loading NaF on PEGylated-AgNPs showed variable cytological effects. Figures 5 and 6 depict the combined influence of NaF@PEG-AgNPs for both fabrication techniques (gallic- and citrate-AgNPs, respectively) on HGFs and OEC cell lines.

For the green synthesis, NaF@PEG gallic-AgNPs revealed an antagonistic effect (combinational index; CI > 1) on fibroblastic lineage despite the significance of decreasing cell viability (*p* < 0.001), which was in close approximation to the pattern of the declining curve of the NaF. Meanwhile, the green nanocomposite exerted a synergistic influence (CI < 1) on epithelial cells, with controversial inhibitory significance when compared with the individual inhibitory effect of PEG-gallic AgNPs and NaF (Fig. 5).

In the chemically synthesized batch, NaF@PEG citrate-AgNPs were more potent in inhibiting cellular metabolic activity, exhibiting an additive effect (CI = 1) in a few combinational doses applied on fibroblastic lineage. The rest of the NaF@PEG citrate-AgNPs combinational doses showed significant synergistic effects (CI < 1) in inhibiting the viability of both cell lines (*p* < 0.001, Fig. 6).

Microbiologically, combining NaF with the PEGylated gallic- and citrate-AgNPs diminished the antimicrobial doses of NaF to ∼ 64 and ∼ 21.3 µg/mL, respectively. Indeed, NaF@PEG gallic-AgNPs revealed an additive antimicrobial effect with FICI = 0.99, where the nanocomposite showed a reduction in the MIC over their separate equivalent anti-*S. mutans* MIC. Meanwhile, adding NaF to PEG citrate-AgNPs records an antagonistic effect of FICI = 5.9, raising the antimicrobial limit of the PEG citrate-AgNPs arm to 32.0 ± 0.0 µg/mL. However, the privilege of decreasing NaF concentration in NaF@PEG citrate-AgNPs gave the needed safe space for the nanocomposite to be cytocompatible, remaining at a much lower dose away from the IC50 of NaF alone (∼ 371 µg/mL and 254 µg/mL for HGFs and OEC, respectively, Table 2).

**Table 2:** Antimicrobial combination and synergy testing results (FICI)

	MIC combination		FICI	Interpretation
	NaF (µg/mL)	PEG-AgNPs (µg/mL)		
NaF@PEG gallic-AgNPs	64.0 ± 0.0	3.3 ± 1.1	0.99	Additive
NaF@PEG citrate-AgNPs	21.3 ± 9.2	32.0 ± 0.0	5.99	Antagonistic

## Discussion

Dental caries is a complex chronic disease that results from the interplay of multiple bacterial strains. Of these bacterial arrays, *S. mutans* is the cornerstone organism responsible for caries initiation [9]. The anti-caries pillars revolve around antibacterial or fluoridated agents. Of the former agents, silver is one of the most commonly used compounds for caries management with proven antibacterial activity [29].

Fluoride, the second anti-caries pillar, is a well-established agent known for its remineralizing properties. It reinforces hydroxyapatite crystals against the carious process with the fluorapatite crystals [2]. However, the corrosive properties of fluoride raise concerns, particularly in the presence of metallic restorations or appliances in the oral cavity. Additionally, fluoride’s reactivity with metals can produce by-products that can exert adverse cytotoxic effects, especially in acidic active carious environments [30, 31]. In the present study, NaF showed a pronounced toxic effect on the primary epithelial and fibroblastic cell lineages, at the high doses (equivalent to 22,600 ppm) down to the recorded IC50 (254 ppm and 371 ppm, respectively). Other studies have documented a similar cytotoxic effect of NaF on human periodontal ligament fibroblasts [32], gingival fibroblasts [33], and promyelocytic leukemia HL-60 cells [34]. Therefore, diminishing fluoride concentration in dental products is a favorable cytocompatibility target.

Combining fluoride with silver is thus an effective strategy in fighting caries and decreasing fluoride levels, where both agents synergistically target cariogenic bacteria. However, the commercially available 38% SDF product contains 44,800 ppm of fluoride [35], a concentration far above the cytological safety limit of 370 and 254 ppm, as reported in the present study. Moreover, there are growing concerns regarding the cytotoxicity of SDF. Investigations have shown that SDF can infiltrate dentin, causing the death of dental pulp cells and gingival fibroblasts, compromising the integrity of the gingival epithelium [36–38]. Therefore, formulations that attain therapeutic benefits while safeguarding oral cellular viability are crucial.

The current study builds its hypothesis on reducing the fluoride dose by loading it on AgNPs, cohering remineralizing properties with the antibacterial activity of the respective elements while keeping safe doses of the nanocomposite. In the current study, we adopted two methods for AgNP synthesis: the chemical citrate-reduction method and the green gallic acid-reduction approach. In both methods, we tuned the relevant factors impacting stability, yielding capacity, and biocompatibility of AgNPs. The green-synthesized AgNPs depicted higher yielding capacity with sharp UV-Vis plasmonic peak with a blue shift, reflected by the smaller size of the nanopoulation over the citrate-reduced AgNPs. Our characterization results align with other reports dealing with the biosynthesis of AgNPs [39, 40]. They have shown that biologically prepared AgNPs revealed high yield, solubility, and stability. However, the small size of gallic acid-AgNPs (6.9 ± 2.1 nm) in the present study raised a future concern about their dislodgement within the dentinal tubules upon translational anti-caries application. Besides size matters, the shape of nanoparticles is highly influential in controlling the biological activity of AgNPs. As far as nano shape is of biological concern, truncated triangular nanoparticles seem more effective and have superior properties [41], a finding reported in citrate-AgNPs with larger safe nano size (63.3 ± 12.6 nm) rather than gallic acid-AgNPs.

The stearic stabilization of gallic-AgNPs and citrate-AgNPs was through polymeric coating using PVP and PEG. Both polymers were of influential stability, especially in citrate-AgNPs, reflected by the boost in yielding capacity and the smooth, narrow, blue-shifted plasmonic UV-Vis peak. Similarly, PVP coating has revealed a matching stearic stability, reducing AgNP size to 16 nm, almost near our TEM results [18]. Also, PEGylation of citrate-AgNPs has wielded a blue shift of the UV-Vis absorbance, albeit with a broader peak and diminished AgNP concentration [17].

Alongside the endorsed stability upon polymeric capping, testing their biocompatibility was crucial as a next step toward clinical implementation of the formulated nanocomposites. Our results on primary oral epithelial and fibroblastic lineages displayed the toxic influence of PVP, reducing IC50 of both AgNP formulae in epithelial cell lineage, attributing to the sensitivity of the epithelial cells over the gingival fibroblasts. Similar to oral epithelial cell perceptiveness, human bronchial epithelial cells (BEAS-2B) have shown apoptotic response to 10 nm sized PVP-AgNPs, pointing out the influence of nano-size in the induction of cytotoxicity [42]. Testing the second surface moieties, PEG revealed consistency in inheriting safety schema, where PEGylation mitigated citrate-AgNP toxicity and boosted the safety silhouette of gallic-AgNPs, confirmed by alleviating the mitochondrial stress seen by MitoTracker Red stain. Likewise, PEGylated-AgNPs have shown a size-dependent safety profile on human keratinocytes, where 30 nm-sized nanoparticles have revealed the minimum impact on cell viability and metabolism [43]. Moreover, PEG toxicity was selective, sensitizing HepG2 liver cancerous cells in a dose-dependent manner [44].

After verifying the compatibility of AgNP formulae, our next step was to validate their potentiality as anti-caries agents by determining their MIC against *S. mutans* together with NaF MIC. Fortunately, NaF was anti-cariogenic at ∼ 170 ppm lower than its IC50 doses, giving a safety range for oral epithelial and gingival fibroblast cells. More promisingly, different formulae of AgNPs (gallic and citrate) showed potent antibacterial activity against *S. mutans* at low dosages, ranging from 5.3 to 10.6 µg/mL. In a similar context, several in vitro and clinical trials document the antibacterial activity of nanosilver against oral bacteria [12, 19]. Experimentally, PVP-chemically reduced-AgNPs, targeting bacterial array responsible for caries and periodontal diseases, have revealed an antibacterial activity between 25 and 50 µg/mL for *S. mutans*,* Streptococcus sanguis*, *Streptococcus mitis*, and *Aggregatibacter actinomycetemcomitans*, and *Fusobacterium nucleatum* [45]. Clinically, PEGylated-AgNPs application to caries lesions has induced a significant double-fold reduction in *S. mutans* counts compared to SDF (21.3% and 10.5%, respectively) [12]. In orthodontic appliances, nanosilver-coated appliances have sustained a decrease in *S. mutans* counts for a month after placement [46]. These results highlight the robustness of nanosilver as a highly effective antibacterial agent for reducing *S. mutans* levels in the oral cavity, qualifying them as nanoplatform for NaF loading.

Nanosilver functionalization with NaF as an anti-cariogenic nanocomposite requires further cytological and microbiological assessments. In our sequential verification, we focused the assays on PEGylated AgNPs as a consistent cytologically safe surface moiety. Our cytological results on gingival fibroblasts revealed that PEGylated AgNPs (PEG gallic- and citrate-AgNPs) counteracted the cytotoxicity of NaF with a combinational antagonistic effect of fluoridated-silver nanocomposites, keeping a safe space for fibroblastic lineage. However, fluoridation of silver nanocomposites (NaF@gallic-AgNPs and NaF@citrate-AgNPs) had synergistic toxicity on oral epithelial cells, sensitizing them at lower doses than their separate dosages. On a microbiological basis, fluoridated green nanocomposite revealed an additive antimicrobial efficacy against *S. mutans.* Surprisingly, NaF@citrate-AgNPs illustrated an antagonistic antimicrobial activity in contrast to other reports, which have displayed a constant synergistic effect of chemically synthesized AgNPs when combined with antibiotics to combat an array of gram-positive and gram-negative strains [47]. Despite the microbiological variations of the fluoridated nanosilver formulae in the current study, reducing NaF concentration provides a protective cytological range.

## Conclusions

In conclusion, nanotechnology in the current study offers dual-purpose anti-cariogenic formulae while preserving cellular viability by adopting fluoridated nanosilver platforms. The nano scaled silver potentiates its antibacterial activity against *S. mutans* while simultaneously reducing the loaded NaF concentrations. Among the synthesis methods studied, gallic-AgNPs revealed superior physiochemical properties and cytocompatibility over citrate-AgNPs. Tuning the surface moiety of both nano formulae *via* PEGylation enhanced the stability and yielding capacity, particularly for citrate-AgNPs, upgrading their safety profile. The antimicrobial propensity and cellular protection favored the PEGylated fluoridated silver nanocomposites, introducing NaF@PEG gallic-AgNPs and NaF@PEG citrate-AgNPs as promising anti-cariogenic platforms.

The optimum safe NaF doses range from 21 µg/mL to 64 µg/mL for PEGylated gallic-AgNPs and PEGylated citrate-AgNPs, respectively. However, the variable microbiological combinational index warrants further optimization to effectively minimize fluoride-associated cytotoxicity while leveraging the antibacterial properties of nanosilver. Furthermore, considering the potential impact of NaF on ionic strength and its effect on the stability of NaF@PEG gallic-AgNPs and NaF@PEG citrate-AgNPs, further research is warranted to enhance nanoplatform stability.

Additionally, clinical translation of these fluoridated nanoplatforms requires extensive preclinical studies and comprehensive long-term clinical trials to investigate the size-dependent influence on the dislodgement of the nanocomposites into the dentinal tubules and its future impact on tooth discoloration. Additionally, probing the consequence of silver ions release on the local oral tissues, with the probability of their implantation in distant organs as previously reported in the lung [48], is crucial for future optimization steps. Form microbiological perspective, clinical implementation necessitates assessing the antibacterial efficacy of these formulations not only against planktonic *S. mutans* but also within biofilm models, simulating the oral environment and caries pathology. Incorporating fluorescence-based viability assays would be crucial to provide a more comprehensive preclinical evaluation of AgNP-mediated antibacterial effects.

## Electronic supplementary material

Below is the link to the electronic supplementary material.

Supplementary Material 1

## Data Availability

The datasets used and/or analysed during the current study are available from the corresponding author on reasonable request.
