# Supplementary material for: Fluoridated silver nanocomposites for caries management: an in-vitro assessment of the cytological and antibacterial profiles
Source: BMC Oral Health. 2025 Mar 9;25:363. doi: 10.1186/s12903-025-05691-2 (PMC11892278; doi:10.1186/s12903-025-05691-2)
Supplement: Supplementary file 1 — Supplementary Material 1 [file 12903_2025_5691_MOESM1_ESM.docx]

**Supplementary material**

**Fluoridated silver nanocomposites for caries management: an in-vitro assessment of the cytological and antibacterial profiles**

Marwa M. Essawy, Samar N. Al Achy, Dalia M. Talaat, Magda M. El-Tekeya, Sara Essa, Nouran Nabil, Nour Ammar

**Results**

**The sturdy antimicrobial efficacy of AgNPs and NaF**

| **AgNPs batch** | **Wavelength (nm)** | **10 mm Absorbance** | **Extinction coefficient (Ɛ)** | **Stock solution (M)** | **Mean MIC (µM)** | **Equivalent MIC (µg/mL)** |
| --- | --- | --- | --- | --- | --- | --- |
| Gallic-AgNPs | 398 | 32.366 | 145 | 0.223 | 49.44 | 5.33 |
| PVP gallic-AgNPs | 419 | 43.338 | 537 | 0.081 | 49.44 | 5.33 |
| PEG gallic-AgNPs | 421 | 46.947 | 537 | 0.087 | 49.44 | 5.33 |
|  |  |  |  |  |  |  |
| Citrate-AgNPs | 470 | 3.9 | 1344 | 0.0029 | 37.08 | 4.0 |
| PVP citrate-AgNPs | 421 | 8.284 | 537 | 0.0154 | 49.44 | 5.33 |
| PEG citrate-AgNPs | 425 | 7.32 | 537 | 0.0136 | 98.88 | 10.66 |
| **Combinational batch** | |  |  |  |  |  |
| NaF |  |  |  |  |  | 64 |
| PEG gallic-AgNPs |  |  |  |  | 30.90 | 3.33 |
|  |  |  |  |  |  |  |
| NaF |  |  |  |  |  | 21.33 |
| PEG citrate-AgNPs |  |  |  |  | 296.65 | 32 |

Supplementary Table 1 displays the detailed results of the equivalent half-maximal cytological and minimal microbiological inhibitory doses of AgNPs in mg/mL with their relevant used extinction coefficient.^1^

**Reference**

1 Paramelle, D. *et al.* A rapid method to estimate the concentration of citrate capped silver nanoparticles from UV-visible light spectra. *Analyst* **139**, 4855-4861, doi:10.1039/c4an00978a (2014).
